# Sex differences in symptom-specific brain abnormalities across disease stages in depression

**DOI:** 10.1186/s13293-026-00964-z

**Published:** 2026-08-04

**Authors:** Xiaoqin Wang, Yi Xia, Junchen Bao, Ciqing Bao, Rui Yan, Zhijian Yao, Qing Lu

**Affiliations:** 1 Department of Psychiatry, the Affiliated Brain Hospital of Nanjing Medical University, Nanjing, 210029 China; 2 Nanjing Brain Hospital, Clinical Teaching Hospital of Medical School, Nanjing University, Nanjing, 210029 China; 3 School of Biological Sciences and Medical Engineering, Southeast University, Nanjing, 210096 China; 4 Child Development and Learning Science, Key Laboratory of Ministry of Education, Nanjing, China; 5 Wenzhou Seventh People’s Hospital, Wenzhou, 325006 China

**Keywords:** Major Depressive Disorder, Sex Difference, Brain Abnormalities, Structural Covariance Network, Structural-Functional Coupling

## Abstract

**Background:**

The incidence and clinical manifestations of major depressive disorder (MDD) differ between sexes. This implies distinct neuropathological mechanisms. This study aimed to investigate sex-specific brain patterns in MDD by stratifying patients according to both sex and disease course.

**Methods:**

We recruited 1170 participants (811 MDD patients and 359 healthy controls) who underwent T1-weighted and resting-state functional magnetic resonance imaging. We explored duration-stage-related, sex-specific differences in gray matter (GM), amplitude of low-frequency fluctuation (ALFF), and structural-functional coupling. We also used the structural covariance network (SCN) to assess morphological connectivity reorganization. Finally, we explored the relationship between key brain measures derived from the preceding multimodal analyses and clinical features.

**Results:**

GM abnormalities in both sexes were observed first appeared in areas supporting sensorimotor and interoceptive functions, and later extended to areas involved in cognitive and emotional processing. Critically, we found distinct sex-specific epicentres of pathology. In male patients, the right postcentral gyrus (PoCG) exhibited early GM atrophy and decreased betweenness centrality within the SCN. In female patients, the left inferior temporal gyrus (ITG) showed early GM atrophy, increased degree centrality within the SCN, reduced ALFF values, and a two-stage evolution of structural-functional coupling with a transition lasting 3 years. Clinically, GM volumes of the right PoCG in male patients were negatively associated with insomnia-early, genital symptoms, and weight loss, while in female patients, GM volumes of the left ITG were negatively associated with depressed mood, work and interest, and psychic anxiety.

**Conclusions:**

This study shows a sex-specific neuropathological origin in MDD, with the left ITG serving as the core pathological area in females and the right PoCG in males. These conclusions not only advance our insight into the sex-dependent mechanisms underlying MDD but also provide a stage-informed framework for individualized interventions, suggesting that the early years of the disease may be a critical period for structure-targeted therapies.

**Supplementary Information:**

The online version contains supplementary material available at https://doi.org/10.1186/s13293-026-00964-z.

## Highlights

Sex-specific hub pathological regions identified: left ITG in females, right PoCG in males.

Atrophy begins in sensorimotor areas and later extends to cognitive regions, following sex-divergent trajectories.

The female left ITG uniquely shows atrophy, increased centrality, reduced ALFF, and a pattern of structural-functional over-coupling.

Male PoCG atrophy correlates with insomnia and somatic symptoms; female ITG atrophy correlates with mood, interest, and anxiety symptoms.

**Supplementary Information:**

The online version contains supplementary material available at https://doi.org/10.1186/s13293-026-00964-z.

## Introduction

Major depressive disorder (MDD) is a prevalent issue that carries heavy medical and economic burdens for individuals and society. Epidemiological research has identified notable sex-related disparities in the prevalence of MDD, with rates of 170.4 per million in females compared to 109.2 per million in males [1]. Females with MDD exhibit greater symptom severity, higher levels of comorbid anxiety, and more atypical depressive symptoms, whereas male patients show a higher prevalence of comorbidity with externalizing behaviours and substance use disorders [2]. Such sex-specific clinical features suggest that the neuropathological features of MDD may differ between males and females across disease stages.

Numerous imaging studies have highlighted sex differences in MDD with respect to brain structure and function. Morphometric magnetic resonance imaging (MRI) studies have reported sex-related differences in the gray matter (GM) volumes of hippocampal [3, 4], amygdala, and anterior cingulate cortex [5, 6] in MDD patients. Another study reported increased GM volumes in the cerebellum and decreased GM volumes in the temporal and prefrontal gyri among male patients, while female patients exhibited decreased GM volumes in the lingual and prefrontal gyri [7]. Additionally, males and females with MDD exhibit distinct magnitudes, laterality, and volumetric and cortical thickness direction in the prefrontal cortex, amygdala, and hippocampus [5, 8]. Beyond structural differences, functional neuroimaging studies using the amplitude of low-frequency fluctuation (ALFF), an index of regional spontaneous brain activity intensity [9], have also revealed sex differences [10]. Our previous work and others have identified sex-specific ALFF differences in the insula, hippocampus, amygdala, caudate nucleus, and orbitofrontal cortex in MDD [11–13].

Despite this growing body of evidence, the reported sex differences have been inconsistent across studies. These inconsistencies may arise from several limitations in previous research. First, most studies have employed unimodal approaches, focusing either on structure or function in isolation, thereby overlooking the multifaceted nature of brain differences in MDD. Given the reciprocal relationship between structure and function, investigating their coupling (e.g., ALFF/GM) is essential for understanding information communication in the brain [17]. Second, most sex-difference studies are cross-sectional, capturing brain changes at a single time point and failing to capture how brain changes unfold across different stages of the illness. As MDD is a chronic illness characterized by recurrent episodes and cumulative burden over time, sex differences observed at one stage may not generalize to others. Third, beyond local regional differences, it remains unknown whether inter-regional anatomical associations exhibit sex-specific patterns over the disease course.

To investigate inter-regional anatomical associations, we constructed structural covariance networks (SCN). Unlike conventional univariate approaches that only detect isolated regional differences, SCN capture coordinated morphological changes across brain regions, revealing how disease-related pathology may propagate along structural pathways and disrupt the normal covariance patterns that reflect neurodevelopmental and ageing processes [14–16]. This network perspective is particularly relevant for MDD, a chronic disorder characterized by progressive impairments in brain morphology and inter-regional coordination [17, 18]. In parallel, to address the other remaining gaps, we employed a multimodal approach integrating structural and functional measures, and stratified patients into four groups based on illness duration to track how brain changes unfold across different phases of MDD.

Here, we bridged these gaps by employing a multimodal, stage-specific framework that integrates: (1) GM volumes for local structural integrity; (2) ALFF values for regional neural activity; (3) SCN for inter-regional anatomical organization; and crucially, (4) structural-functional coupling (ALFF/GM) to capture their interplay. Patients were categorized into four stages of disease course (onset: 0–3 months; short-term: 4–12 months; mid-term: 1–3 years; long-term: >3 years). We sought to delineate sex-specific patterns of these multimodal features across different phases of MDD. We hypothesized that male and female patients would exhibit distinct patterns of brain differences, both locally and at the network level, that are stage-dependent. We hope to provide a neurobiological framework for understanding the clinical dimorphism observed in MDD.

## Methods and materials

### Participants

Between February 2011 and December 2023, 818 patients with MDD were enrolled in the study from the Affiliated Nanjing Brain Hospital of Nanjing Medical University, and 360 healthy controls (HC), matched for age and sex, were recruited from the community. The course of the patient’s total illness was evaluated by two senior psychiatrists through using the Mini International Neuropsychiatric Interview (M.I.N.I.). Specific inclusion and exclusion criteria for all participants are provided in the Supplementary “1.1 Data selection”.

### MRI scan acquisitions and pre-processing

Imaging data were acquired using a 3-Tesla Siemens Verio scanner with an eight-channel radio frequency coil at Nanjing Brain Hospital. Scanning parameters can be found in the Supplementary section “1.2 Data scan acquisitions”.

High-spatial-resolution T1-weighted MRI data were processed using standard voxel-based morphometry through the Computational Anatomy Toolbox 12 (CAT12; http://dbm.neuro.unijena.de/cat12/) within the Statistical Parametric Mapping 12 (SPM12; http://www.ffl.ion.ucl.ac.uk/spm/) software. Subsequently, the Data Processing Assistant for Resting-State fMRI (DPARSF; http://rfmri.org/DPARSF) was employed to analyse the functional imaging data [19], using parameters consistent with those used in previous studies [13, 20]. For the specific steps of GM and ALFF image acquisition, see the Supplementary “1.3 Data pre-processing” section. Next, both GM and ALFF images were parcellated into 166 regions using the Automated Anatomical Labelling (AAL) 3 atlas [21]. Finally, for each participant and each region, mean GM volumes and mean ALFF values were extracted for subsequent analysis.

Participants were excluded if their head rotation exceeded 3°, head translation surpassed 3 mm, or the interquartile range of normalized GM intensity was below 0.8, indicating insufficient image uniformity. After excluding 7 MDD patients and 1 HC due to excessive head motion or poor image quality, the final sample comprised 811 patients with MDD (326 males, 485 females) and 359 HC (138 males, 221 females).

### Statistical analysis

#### Demographic statistics

Demographic and clinical characteristics were analysed using Statistical Product and Service Solutions (SPSS, version 25.0). For statistics analysis, age differences, educational levels, and total intracranial volume (TIV) among four groups (male MDD, male HC, female MDD, and female HC) at four duration stages, one-way analysis of variance (ANOVA) was performed. Additionally, a two-sample t-test was used to compare illness duration, number of episodes, and 17-item Hamilton Depression Rating Scale (HAMD_17_) total scores between male and female MDD. *P* < 0.05 was considered significant.

#### GM analysis: stage-specific dynamic patterns in MDD

We implemented stage-specific methodologies to investigate stage-related patterns of GM volume atrophy in MDD. According to the distribution of the data we collected, participants were categorized into four subgroups based on the total duration of MDD: 0–3 months (male/female: 55/74), 3–12 months (male/female: 95/131), 1–3 years (male/female: 78/126), and over 3 years (male/female: 98/154).

To account for potential confounding by age and sex, we performed independent matching procedures for each patient subgroup. Specifically, the MDD patients were divided into eight subgroups based on sex (male/female) and disease stage (0–3 months, 3–12 months, 1–3 years, > 3 years). For each of these eight subgroups, we selected a matched subset of HC from the overall HC pool (male HC: *N* = 138, female HC: *N* = 221) based on exact sex match and age within ± 3 years. For each patient, we first identified all HC candidates of the same sex whose age fell within this ±3-year range; among those eligible candidates, we then selected the one with the closest age to the patient (nearest-neighbour matching without replacement) to form the matched pair. Due to sample limitations, some HC participants were included in more than one matched control set. This approach ensured that each patient subgroup was compared against a control group with comparable sex and age distributions.

To examine stage-related changes in GM volumes, we performed a two-sample t-test comparing GM volumes between MDD patients and HC, separately for males and females, at each of the four illness stages (a total of 8 comparisons).

To determine whether the patterns of GM differences differed between sexes, a two-way ANOVA was conducted at each stage, with sex (male/female) and group (MDD/HC) as fixed factors, allowing us to assess their main effects and interaction.

All statistical analyses were performed in SPM12. For each two-sample t-test and two-way ANOVA, statistical significance was determined using Gaussian Random Field (GRF) correction with a voxel-level threshold of *p* < 0.005 and a cluster-level threshold of *p* < 0.05. To account for multiple comparisons across the eight two-sample t-tests, we applied an additional Bonferroni correction, setting the statistical significance at cluster-level *p* < 0.05/8 = 0.00625. TIV and years of education were included as covariates in all analyses.

#### Structural covariance network analysis

To construct SCN, regional GM volumes were extracted from 166 regions defined by the AAL3 and adjusted for TIV and years of education. The residualized GM values were then used to construct group-level correlation matrices separately for each subgroup. To ensure sufficient statistical power for graph-theoretical analyses, patients and controls were compared at the full-sample level, i.e., 326 male MDD vs. 138 male HC, and 485 female MDD vs. 221 female HC, without further stage stratification.

To enable case-control comparisons, each correlation matrix was threshold into a binarized network across a range of sparsity thresholds (male: 0.14–0.42; female: 0.14–0.50; with a step of 0.01). For each threshold, we computed seven global metrics (clustering coefficient, characteristic path length, gamma, lambda, sigma, global efficiency, local efficiency) and three nodal metrics (degree centrality, betweenness centrality, nodal efficiency). The details of the SCN construction and sparsity threshold selection could be seen in Supplementary “1.4 Construction of SCN and threshold selection”.

To test for significant differences between patient and control groups, we employed nonparametric permutation tests [22]. Specifically, we performed 1000 permutation iterations. In each iteration, we randomly reassigned each participant’s residualized GM values to either the patient or control group (separately for males and females), recomputed the group-level correlation matrices and network metrics for each randomized grouping, and recorded the between-group difference for each metric. This generated a null distribution of 1000 between-group differences for each metric. The observed between-group difference was then compared against this full null distribution (not against the mean of the 1000 permutations), and the p-value was calculated as the proportion of permuted differences that exceeded the observed absolute difference (two-tailed). For normalized metrics (gamma, lambda, sigma), 100 random networks were generated per threshold (using the randmio_und function, which preserves degree distribution) to obtain the expected values for random networks; this step is separate from the permutation testing and is used only for normalization. Statistical significance was determined at *p* < 0.05 after false discovery rate (FDR) correction.

#### ALFF analysis: stage-specific dynamic trajectory patterns in MDD

Following the same stage-specific framework and case-control matching as described for GM analysis, we performed a two-sample t-test and a two-way ANOVA to examine stage-related ALFF differences in MDD across four illness stages. Statistical significance was determined following the same procedure as in the GM analysis (see Sect. "GM analysis: stage-specific dynamic patterns in MDD"). Years of education were included as a covariate.

#### Structural-functional coupling analysis: stage-specific dynamic trajectory patterns in MDD

We used the regions identified from the stage-specific GM and ALFF analyses and examined coupling within these regions at each stage, applying the same stage- and sex-specific grouping used in the GM/ALFF analyses. After obtaining each subject’s ALFF and GM maps as described in Sect. "MRI scan acquisitions and pre-processing", we assessed structural-functional coupling by computing ALFF-to-GM ratios. Regional coupling values were then extracted for each of the 166 regions defined by the AAL3 atlas. To investigate whether structural-functional coupling differs in regions with structural or functional changes, we focused on regions previously identified as showing sex-specific correlations with illness duration (as reported in Sect. " Stage-specific GM dynamic patterns in MDD" and "Stage-specific ALFF dynamic patterns in MDD"). In these regions, we compared coupling values between MDD patients and their matched HC using general linear models, with TIV and years of education as covariates. These analyses were performed separately for males and females within each of the four illness stages. Multiple comparisons across the selected regions were corrected using the FDR method, with *p* < 0.05 as the significance threshold.

#### Correlations analysis

To examine the clinical significance of the identified brain measures (i.e., GM, ALFF, SCN, and their coupling) in MDD patients, we performed Spearman correlation analyses between these measures and each HAMD_17_ item score within the MDD group, separately for each sex. Normality tests were conducted prior to the analyses, and statistical significance was set at FDR-corrected *p* < 0.05.

## Results

### Demographic and clinical characteristics

There were no statistically significant differences between MDD and HC at each stage concerning age and TIV. HC exhibited significantly higher education level than MDD patients. Additional information was provided in Tables 1 and 2. Given that the groups included in the SCN analysis were inconsistent with those in the main analysis, we have additionally provided demographic information, which can be found in Table 3.

**Table 1:** The Demographic and clinical characteristics

Variables		0–3 months		4–12 months		1–3 years		> 3 years	
		MDD	HC	MDD	HC	MDD	HC	MDD	HC
**Sex**	Male	55	55	95	95	78	78	98	98
	Female	74	74	131	131	126	126	154	154
**Age**,** y**	Male	26.16 ± 9.84	26.45 ± 8.8	28.14 ± 10.44	28.61 ± 9.31	27.47 ± 10.33	27.69 ± 9.24	30.36 ± 9.70	30.19 ± 9.21
	Female	27.82 ± 9.86	27.55 ± 9.09	27.91 ± 10.57	27.95 ± 9.45	25.33 ± 8.98	25.79 ± 7.63	29.18 ± 10.08	28.74 ± 9.15
**Education**,** y**	Male	13.76 ± 3.13	15.15 ± 1.91	14.11 ± 2.81	15.24 ± 2.00	13.97 ± 2.87	15.13 ± 2.02	14.04 ± 2.62	15.50 ± 2.13
	Female	13.32 ± 2.75	15.19 ± 1.83	13.78 ± 2.85	15.20 ± 2.01	13.56 ± 2.54	15.48 ± 1.77	13.41 ± 2.71	15.44 ± 1.97
**TIV**	Male	1553 ± 150	1558 ± 126	1553 ± 114	1553 ± 119	1572 ± 112	1547 ± 119	1551 ± 114	1558 ± 115
	Female	1397 ± 88	1402 ± 119	1400 ± 91	1465 ± 133	1405 ± 96	1416 ± 109	1403 ± 94	1402 ± 114
**Total duration of illness**,** mo**	Male	2.06 ± 0.76		8.70 ± 3.24		25.46 ± 6.92		81.47 ± 49.17	
	Female	1.99 ± 0.81		8.71 ± 3.13		26.67 ± 6.85		89.66 ± 57.86	
**Depression**, **No. of episodes**	Male	1.09 ± 0.28		1.49 ± 0.90		2.28 ± 2.20		2.78 ± 2.25	
	Female	1.08 ± 0.28		1.56 ± 1.21		2.18 ± 1.92		2.97 ± 2.46	
**HAMD**_**17**_ **total scores**	Male	20.60 ± 6.37		22.80 ± 5.20		23.78 ± 3.96		26.23 ± 6.94	
	Female	21.97 ± 5.56		23.82 ± 5.65		23.95 ± 5.02		25.92 ± 6.68	
**Marriage (unmarried/married/divorced)**	Male	38/16/1	40/15/0	63/31/1	60/35/0	54/22/2	50/28/0	53/43/2	54/43/1
	Female	43/30/1	48/26/0	78/52/1	88/43/0	90/32/4	100/26/0	85/63/6	98/55/1
**Family history (no/yes)**	Male	50/5	55/0	78/17	95/0	66/12	78/0	80/18	98/0
	Female	65/9	74/0	112/19	131/0	104/22	126/0	123/31	154/0

MDD, major depressive disorder; HC, health controls; TIV, total intracranial volume; HAMD_17_, 17-item of Hamilton Depression Rating Scale; y, year; mo, month

Data were presented as the range of minimum-maximum (mean ± SD)

**Table 2:** The matching distributions in sex and age

Variables			MDD	HC	t	*p*
**0–3 months**	**Age**,** y**	Male	26.16 ± 9.84	26.45 ± 8.8	-0.163	0.871
		Female	27.82 ± 9.86	27.55 ± 9.09	0.173	0.863
	**Education**,** y**	Male	13.76 ± 3.13	15.15 ± 1.91	-2.79	0.006
		Female	13.32 ± 2.75	15.19 ± 1.83	-4.853	< 0.001
	**TIV**	Male	1553 ± 150	1558 ± 126	-0.165	0.869
		Female	1397 ± 88	1402 ± 119	-0.301	0.764
**4–12 months**	**Age**,** y**	Male	28.14 ± 10.44	28.61 ± 9.31	-0.330	0.742
		Female	27.91 ± 10.57	27.95 ± 9.45	-0.031	0.975
	**Education**,** y**	Male	14.11 ± 2.81	15.24 ± 2.00	-3.212	0.002
		Female	13.78 ± 2.85	15.20 ± 2.01	-4.659	< 0.001
	**TIV**	Male	1553 ± 114	1553 ± 119	-0.024	0.981
		Female	1400 ± 91	1465 ± 133	-0.043	0.966
**1–3 years**	**Age**,** y**	Male	27.47 ± 10.33	27.69 ± 9.24	-0.139	0.890
		Female	25.33 ± 8.98	25.79 ± 7.63	-0.431	0.667
	**Education**,** y**	Male	13.97 ± 2.87	15.13 ± 2.02	-2.909	0.004
		Female	13.56 ± 2.54	15.48 ± 1.77	-6.934	< 0.001
	**TIV**	Male	1572 ± 112	1547 ± 119	1.349	0.179
		Female	1405 ± 96	1416 ± 109	-0.851	0.396
**> 3 years**	**Age**,** y**	Male	30.36 ± 9.70	30.19 ± 9.21	0.121	0.904
		Female	29.18 ± 10.08	28.74 ± 9.15	0.397	0.692
	**Education**,** y**	Male	14.04 ± 2.62	15.50 ± 2.13	-4.281	< 0.001
		Female	13.41 ± 2.71	15.44 ± 1.97	-7.542	< 0.001
	**TIV**	Male	1551 ± 114	1558 ± 115	-0.429	0.669
		Female	1403 ± 94	1402 ± 114	0.121	0.904

MDD, major depressive disorder; HC, health controls; TIV, total intracranial volume; y, year

Data were presented as the range of minimum-maximum (mean ± SD)

**Table 3:** The Demographic and clinical characteristics for SCN

Variables		MDD	HC	t	*p*
Male	N	326	138		
	Age, y	28.31 ± 10.16	29.13 ± 8.56	-0.829	0.408
	Education, y	14.00 ± 2.81	15.36 ± 2.03	-5.131	< 0.001
	TIV	1557 ± 119.96	1549 ± 114.97	0.644	0.520
Female	N	485	221		
	Age, y	27.63 ± 9.99	27.86 ± 7.95	-0.297	0.767
	Education, y	13.54 ± 2.71	15.64 ± 2.01	-10.331	< 0.001
	TIV	1401 ± 92.57	1401 ± 112.50	0.084	0.933

SCN, Structural covariance network; MDD, major depressive disorder; HC, health controls; TIV, total intracranial volume; y, year

Data were presented as the range of minimum-maximum (mean ± SD)

### Stage-specific GM dynamic patterns in MDD

**Fig. 1:**
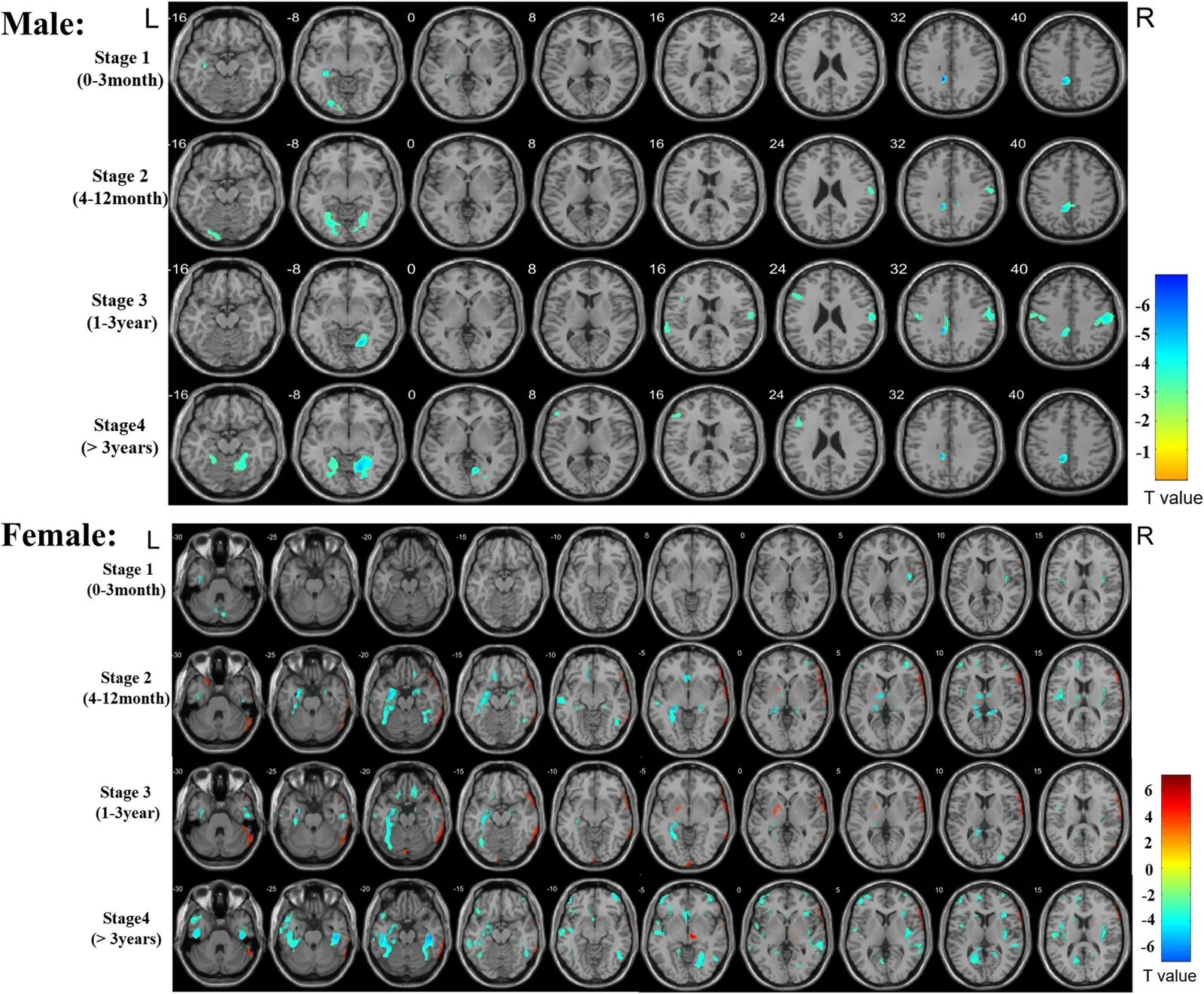
Stage-specific GM abnormality patterns in male and female patients with MDD. In the colorbar, blue indicates that the GM of MDD is smaller than that of HC, and red indicates an increase

Male patients showed no increased GM volume in any region compared to the HC group across all stages. Stage-related GM atrophy differences were observed with increasing illness duration. At stage 1 (*n* = 52; 2.06 ± 0.76 months), atrophy was localized to the left middle cingulum gyrus (MCG), the right postcentral gyrus (PoCG), and the left lingual gyrus. With later stages, atrophy was additionally observed in the right lingual gyrus at stage 2 (*n* = 95; 8.70 ± 3.24 months), the left inferior frontal gyrus (IFG) pars triangularis at stage 3 (*n* = 78; 25.46 ± 6.92 months), and further involved frontal cortical regions (IFG pars triangularis and orbitalis, right middle frontal gyrus [MFG] pars orbitalis) by stage 4 (*n* = 98; 81.47 ± 49.17 months). The stage distribution of these atrophic regions is summarized in Fig. 1 and detailed in Supplementary Table S1.

Female MDD patients exhibited a more extensive pattern of GM atrophy. At stage 1 (*n* = 74; 1.99 ± 0.81 months), atrophy involved the left inferior temporal gyrus (ITG) and bilateral insula. At stage 2 (*n* = 131; 8.71 ± 3.13 months), abnormalities extended throughout the limbic system (left putamen, bilateral hippocampus) and temporal cortex (bilateral ITG, bilateral middle temporal gyrus [MTG], right superior temporal gyrus [STG]), with additional involvement of frontal regions (IFG pars triangularis and orbitalis, right MFG, left superior frontal gyrus [SFG]). At stage 3 (*n* = 126; 26.67 ± 6.85 months), occipital regions became affected (left calcarine, left precuneus, right middle occipital gyrus [MOG]). By stage 4 (*n* = 154; 89.66 ± 57.86 months), atrophy involved all major cortical regions, with additional parietal involvement (bilateral PoCG, right superior parietal gyrus [SPG]).

In addition to the GM atrophy described above, we also observed several regions with increased GM volumes in female MDD patients compared to HC at certain stages (e.g., right MTG and right STG at stage 2). However, these increases were less consistent across stages and were therefore not the primary focus of our analysis. A complete list of all regions with significant GM differences (both increased and decreased) is provided in Supplementary Table S2. The stage distribution of GM abnormality in female patients is illustrated in Fig. 1 and detailed in Supplementary Table S2.

The results for the sex-by-group interaction of GM among the four groups (MDD male, MDD female, HC male, HC female) were shown in Supplementary 2.1 section and Table S3. Furthermore, we extracted the GM values of several hub brain regions to illustrate the changes at different stages. Please refer to Supplementary Material Figure S1 for details.

### Altered structural covariance network in MDD

The results of the global topology metrics in SCN were shown in Supplementary 2.3 and Table S4.

Regarding regional topology metrics, male patients with MDD showed increased degree centrality frontal gyrus (left medial and bilateral posterior orbital frontal cortex [OFC]) and right MCG), occipital gyrus (right lingual and bilateral fusiform) and bilateral nucleus accumbens (Supplementary Table S5); simultaneously, they exhibited decreased betweenness centrality in some regions of the frontal cortex (right IFG pars opercularis and orbitalis, left medial SFG, the right anterior OFC, right PoCG, and left cuneus (Supplementary Table S6).

Female patients with MDD displayed increased degree centrality in some regions of the frontal cortex (bilateral dorsolateral SFG, bilateral MFG, right IFG pars orbitalis, left medial SFG, right ventromedial prefrontal cortex, left posterior OFC, and pregenual anterior cingulum gyrus), and temporal cortex (right MTG and bilateral ITG) (Supplementary Table S5). They also exhibited decreased betweenness centrality in some regions of the frontal cortex (left IFG pars opercularis, right posterior OFC, right inferior parietal gyrus [IPG] and temporal cortex (right STG and bilateral STP) (FDR-corrected *p* < 0.05; Supplementary Table S6).

### Stage-specific ALFF dynamic patterns in MDD

**Fig. 2:**
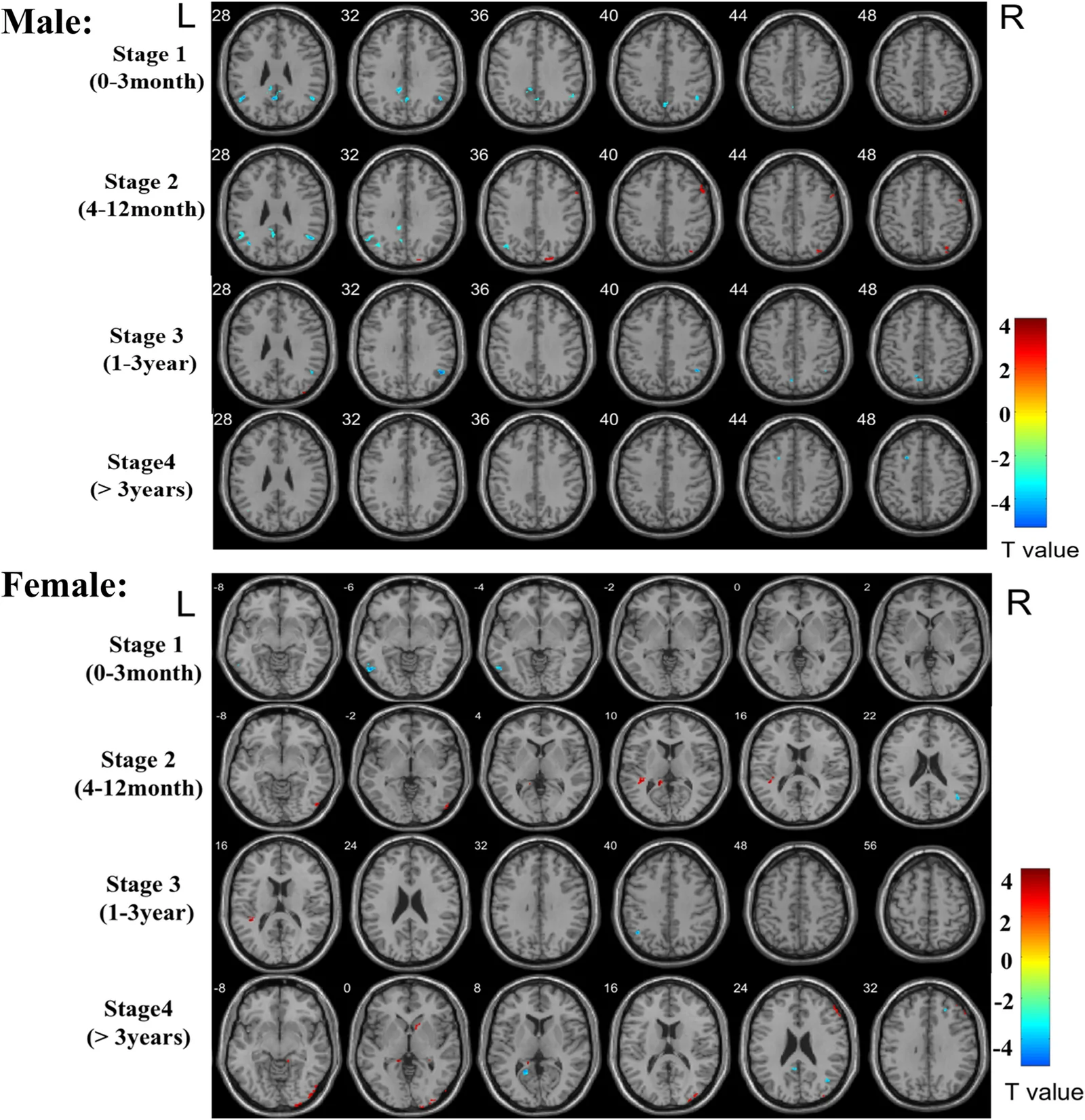
Stage-specific ALFF abnormality patterns in male and female patients with MDD. In the colorbar, blue indicates that the GM of MDD is smaller than that of HC, and red indicates an increase

Male MDD patients exhibited stage-related ALFF differences across disease stages, with both increases and decreases in different brain regions. At stage 1, ALFF values were reduced in the left MCG, the bilateral angular gyrus, and the bilateral precuneus, while increased in the right superior occipital gyrus (SOG). At stage 2, ALFF abnormalities extended to frontal and occipital cortices, with increased ALFF observed in the parietal cortex (right precentral gyrus and right SPG). By stage 3, ALFF differences were predominantly localized to the parietal cortex (left precentral gyrus and right IPG) and the occipital cortex (right MOG and right angular). At stage 4, ALFF values decreased in the left SFG and left angular, while increasing in the right MOG. The stage distribution of ALFF abnormality in male patients is illustrated in Fig. 2 and detailed in Supplementary Table S7.

Female MDD patients showed stage-related ALFF differences with increasing illness duration. At stage 1, decreased ALFF was observed in the left ITG. ALFF abnormalities involved multiple regions: increased ALFF in the left STG, left inferior occipital gyrus (IOG), and left hippocampus, with decreased ALFF in the right MOG. At stage 3, increased ALFF was observed in the left STG, while decreased ALFF was noted in the left angular gyrus. By stage 4, extensive ALFF dysfunction was observed across the temporal cortex (right ITG and right MTG), frontal cortex (right IFG pars triangularis and right MFG), parietal cortex (left precentral gyrus and right IPG), limbic system (right caudate, bilateral hippocampus and right parahippocampal), and occipital cortex (right IOG, right MOG). Statistical thresholds were set at GRF-corrected voxel-level *p* < 0.001 and cluster-level *p* < 0.05. The stage distribution of ALFF abnormality in female patients is illustrated in Fig. 2 and detailed in Supplementary Table S8.

The results for the sex-by-group interaction of ALFF were shown in Supplementary 2.2 section and Table S9.

### Structural-functional coupling

In male patients, no significant coupling differences were observed in either GM-altered or ALFF-altered regions across disease stages, whereas female patients exhibited stage-related coupling abnormalities across disease stages.

In female patients, we examined structural-functional coupling (ALFF-to-GM ratios) within regions that showed GM or ALFF differences at each disease stage. Within GM-altered regions, increased coupling values were observed in the bilateral insula and bilateral hippocampus at stage 2. At stage 3, additional regions showing increased coupling included the temporal gyrus (right STG and bilateral ITG) and left IFG pars orbitalis. By stage 4, increased coupling extended across a wide range of regions, including the frontal cortex (right dorsolateral SFG, bilateral MFG, bilateral precentral gyrus), temporal cortex (bilateral STG, left STP, left MTG, and bilateral ITG), limbic system (bilateral insula, left caudate), and right fusiform gyrus.

**Fig. 3:**
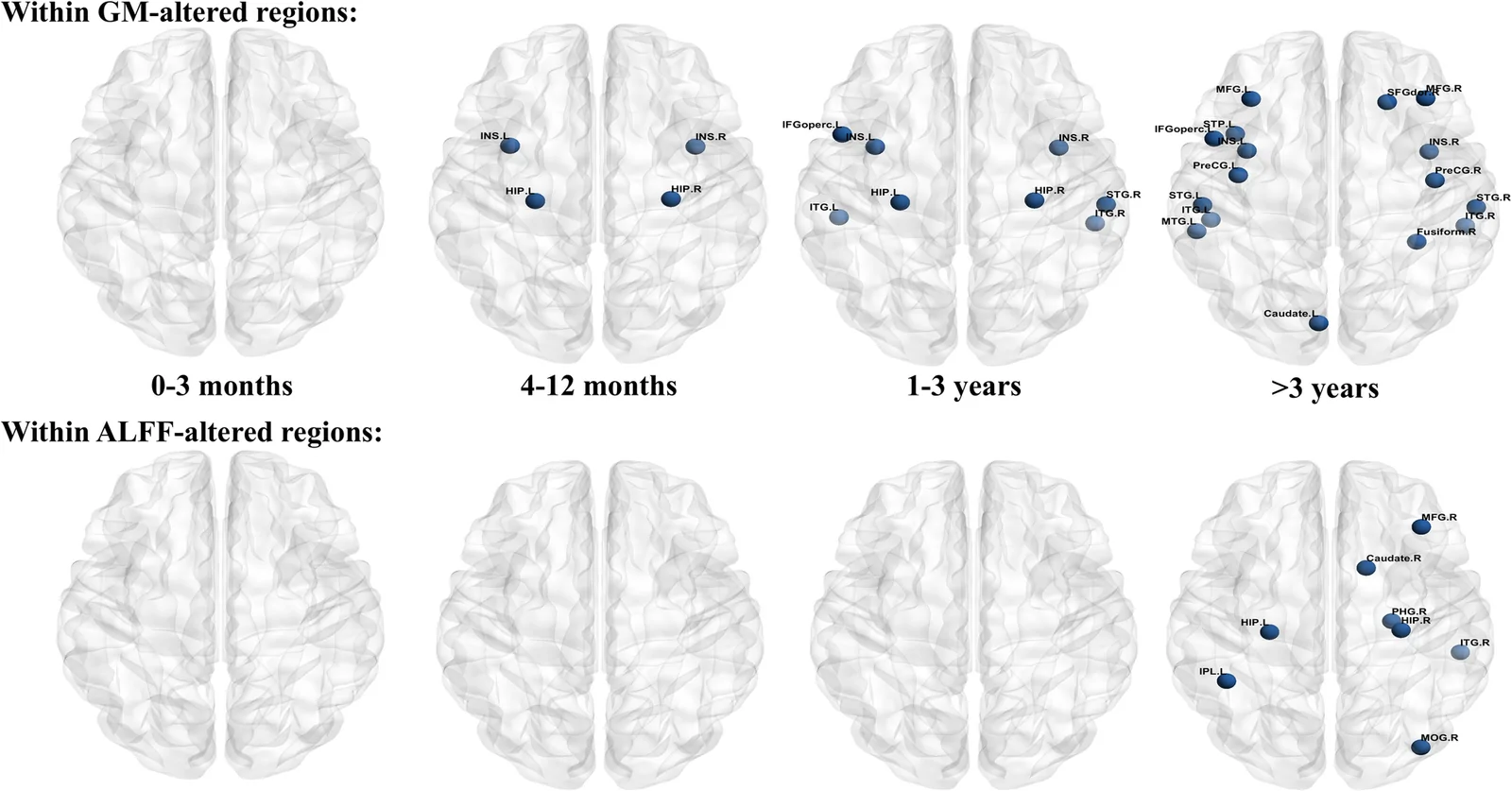
Stage-specific structural-functional coupling differences in female MDD patients. Abbreviations: GM, gray matter; ALFF, amplitude of low-frequency fluctuation; IFGoperc, left inferior frontal gyrus pars opercularis; HIP, hippocampus; INS, insula; IPL, inferior parietal lobule; ITG, inferior temporal gyrus; MFG, middle frontal gyrus; MOG, middle occipital gyrus; MTG, middle temporal gyrus; PHG, parahippocampal gyrus; PreCG, precentral gyrus; SFG, superior frontal gyrus; SFGdor, superior frontal gyrus dorsal part; STG, superior temporal gyrus; STP, superior temporal pole; L, left; R, right

Within ALFF-altered regions, no abnormal coupling was observed during the first three disease stages. Increased coupling was only observed at stage 4, involving the right MFG, right ITG, bilateral hippocampus, right parahippocampal, right caudate, right MOG, and left inferior parietal gyrus (FDR-corrected *p* < 0.05, Fig. 3).

### Correlations with clinical symptoms

Spearman correlation analyses were performed to examine the relationships between the GM volumes of the right PoCG in males and the left ITG in females and each HAMD_17_ item score.

**Fig. 4:**
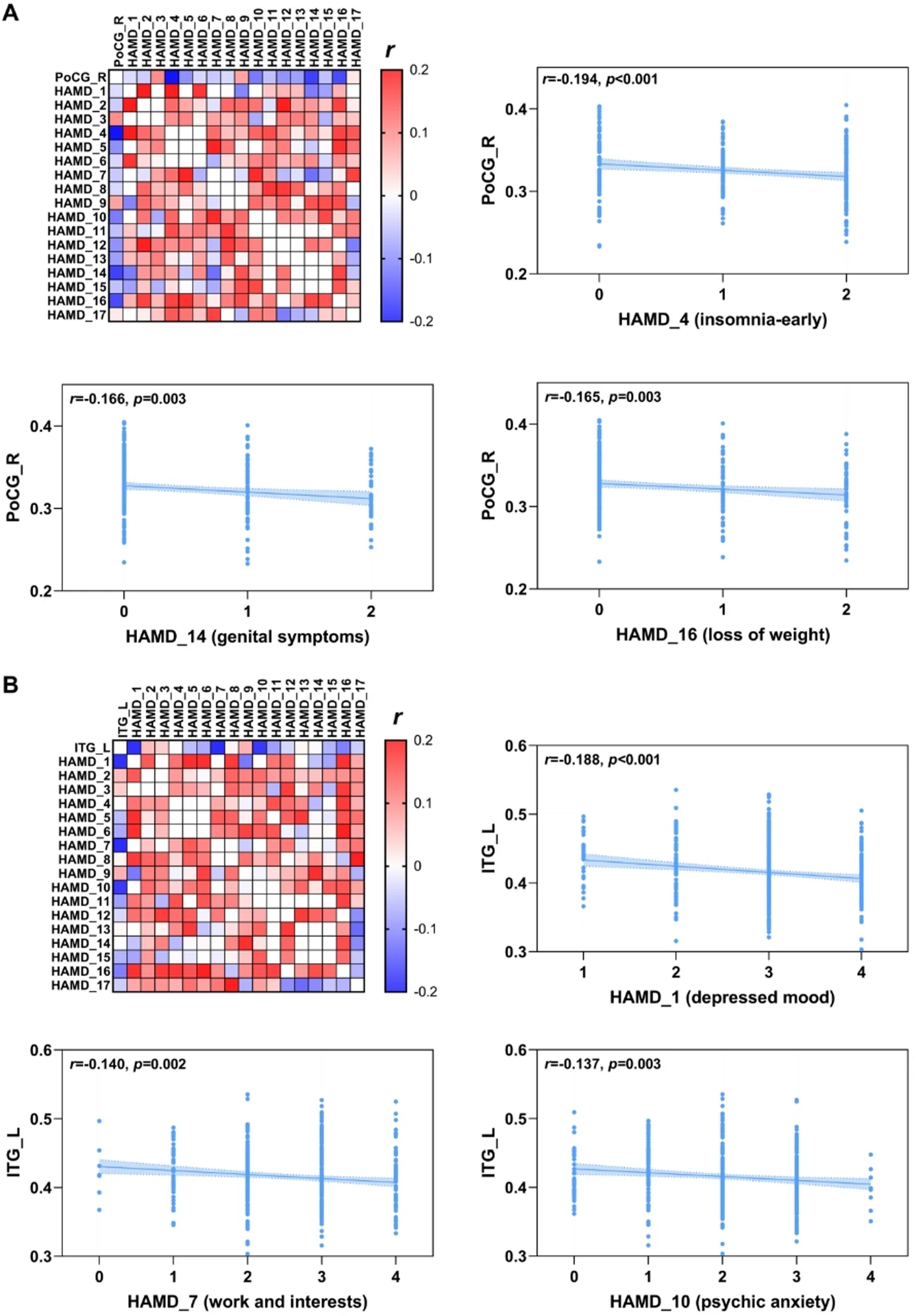
Correlations between GM volume and individual HAMD_17_ item scores. (**A**) Male patients; (**B**) Female patients. Abbreviations: PoCG_R, right postcentral gyrus; ITG_L, left inferior temporal gyrus; HAMD_17_, 17-item Hamilton Depression Rating Scale. All correlations were FDR-corrected *p* < 0.05

In males, the GM volumes of the right PoCG were significantly correlated with HAMD_17_ item 4 (insomnia-early, *r* = -0.194, FDR-corrected *p* < 0.001), item 14 (genital symptoms, *r* = -0.166, FDR-corrected *p* = 0.003), and item 16 (loss of weight, *r* = -0.165, FDR-corrected *p* = 0.003) (Fig. 4A).

In females, the GM volumes of the left ITG showed significant correlations with HAMD_17_ item 1 (depressed mood, *r* = -0.188, FDR-corrected *p* < 0.001), item 7 (work and interest, *r* = -0.140, FDR-corrected *p* = 0.002), and item 10 (psychic anxiety, *r* = -0.137, FDR-corrected *p* = 0.003) (Fig. 4B).

No significant correlations were observed with other HAMD_17_ items.

## Discussion

This study found that GM differences in both sexes were observed first in regions supporting sensorimotor and interoceptive functions (precentral, insular, and temporal gyrus) [23] and subsequently extended to areas involved in cognitive and emotional processing (frontal, parietal, occipital gyrus, and limbic system) [24]. Critically, we revealed a fundamental sex difference in the neurobiological substrates of MDD: male patients exhibited early structural abnormalities in the right PoCG and left MCG, whereas female patients showed initial involvement of the left ITG and bilateral insula. These distinct epicentres are associated with opposing patterns of network centrality, which may determine their capacity to propagate pathology across the brain.

We found that male patients exhibited specific atrophy of the right PoCG and left MCG compared to female patients throughout the disease course. The PoCG, a primary hub of the sensorimotor network, is central to bodily awareness and psychomotor function [25]. Its early involvement in male patients is consistent with prior reports of male-specific sensorimotor network differences in MDD [26, 27]. Our previous research also demonstrated that MDD patients with abnormalities in the sensorimotor network present with psychomotor symptoms [28, 29]. Clinically, we found that GM of the right PoCG in male patients was negatively associated with early insomnia, genital symptoms, and weight loss, which implicates disrupted sensorimotor and autonomic regulation. Therefore, we support the notion that sensorimotor dysfunction is a core feature of male MDD, manifesting as somatic and psychomotor symptoms.

Additionally, the right PoCG showed decreased betweenness centrality, indicating that this region becomes less integrated with other brain networks [16]. Rather than serving as a “source” that propagates pathology to distant regions, the PoCG appears to function as an “isolated lesion”. This interpretation is supported by the stage-related pattern of GM atrophy in males: structural and functional differences remained relatively confined throughout the disease course, without the widespread expansion that was observed in females.

Female patients exhibited early GM atrophy in the bilateral insula, a region that integrates interoceptive signals and mediates emotional awareness [30, 31]. Insular abnormalities are known to underlie somatic symptoms such as gastrointestinal distress and nausea [32], which are disproportionately reported by depressed females [33]. The persistence of insular atrophy throughout the disease course in female patients may therefore explain the higher burden of somatic symptoms in this population.

Notably, we also identified the left ITG as a key epicentre in females, as supported by multimodal evidence: it showed early GM atrophy, increased degree centrality within the SCN, and reduced ALFF values at early stages, together with a two-stage pattern of structural-functional coupling. Previous studies have reported that the ITG is interconnected with other brain regions, including the frontal, parietal, temporal, occipital, and limbic regions, and is crucial for visual object recognition, language comprehension, and emotion regulation [34, 35]. Female patients exhibited an increased degree centrality of the left ITG, indicating that this region becomes hyperconnected within the sensorimotor network. This heightened connectivity may enable the ITG to act as a “propagating hub”, facilitating the spread of pathology to interconnected brain regions. Consistent with this interpretation, female patients showed stage-related GM atrophy that was observed from the ITG and insula to frontal, temporal, parietal, occipital, and limbic regions at later stages. Clinically, GM of the left ITG was negatively associated with depressed mood, work and interest, and psychic anxiety symptoms that represent the “classic” affective-cognitive presentation of MDD. This pattern aligns with clinical observations that female patients more frequently present with widespread affective and cognitive symptoms, whereas male patients often present with localized somatic complaints [2, 36].

The most striking sex difference in our study, however, was not in individual brain regions, but in the stage-related patterns of structural-functional integration. While both sexes exhibited structural-functional asynchrony, with functional differences preceding structural changes in certain regions, consistent with previous studies [37], the stage-related patterns of coupling revealed a striking sex difference. Male patients showed no significant differences in structural-functional coupling compared to healthy males at any disease stage, suggesting that structural and functional differences remain relatively “in sync” throughout the disease course.

In contrast, female patients exhibited a stage-dependent pattern of coupling abnormalities. During the first three years of illness, abnormal coupling was driven predominantly by structural differences, with functional abnormalities playing a secondary role. After three years, however, the aberrant ALFF-to-GM relationship arose from the interplay between structure and function, marking a phase shift from “structure-led” to “structure-function interactive” pathology. This two-stage model has important clinical implications. The first three years may represent a “window of opportunity” during which interventions targeting structural integrity could prevent or delay the emergence of more widespread dysfunction. Conversely, the post-three-year stage, characterized by interactive pathology, may require combined strategies addressing both structural and functional deficits.

### Limitation

This study still has some shortcomings that need to be considered. Firstly, some of the patients were taking medications in our study, so the effect of treatment regimens cannot be completely ruled out. Secondly, the functional pattern was only evaluated for local anatomical areas, but large-scale inter-regional communication might also be considered. Third, MDD is a clinically and biologically heterogeneous disorder, and we did not consider the influence of different subtypes with inherently different neurobiological profiles. This risk is misinterpreting subtype differences as stage-related patterns. Forth, as described in the Methods section, some healthy control participants were included in more than one patient subgroup comparison. This reuse of HC participants introduces statistical non-independence across comparisons, which may slightly increase the risk of type I error. Lastly, the cross-sectional design of this study cannot directly establish the real-world temporal sequence of illness progression, as it captures group differences at discrete stages rather than following the same individuals over time. Thus, longitudinal studies should be performed to further clarify the relationship between structure and function across the illness’s progression.

## Conclusion

In summary, this study delineates a sex-specific framework for understanding the neurobiological progression of MDD. We demonstrate that male patients are characterized by a “confined” pathology, anchored by an isolated epicentre (PoCG) with decreased network centrality, manifesting as localized somatic symptoms and lacking progressive coupling abnormalities. In contrast, female patients exhibit a “propagating” pathology, driven by a hyperconnected epicentre (ITG) that acts as a “hub,” leading to widespread cortical involvement and a distinct two-stage evolution of structural-functional coupling. These findings not only advance our understanding of the sex-dependent mechanisms underlying MDD but also offer a temporal roadmap for personalized interventions, suggesting that the early years of the disease may be a critical period for structure-targeted therapies.

## Supplementary Information

Below is the link to the electronic supplementary material.

Supplementary Material 1

## Data Availability

No datasets were generated or analysed during the current study.
