# Supplementary material for: Sex differences in symptom-specific brain abnormalities across disease stages in depression
Source: Biol Sex Differ. 2026 Aug 4;17:174. doi: 10.1186/s13293-026-00964-z (PMC13726080; doi:10.1186/s13293-026-00964-z)
Supplement: Supplementary file 1 [file 13293_2026_964_MOESM1_ESM.docx]

**Online Supplementary Materials**

Table of contents

[1 Supplementary method	1](#_Toc231769246)

[1.1 Data selection	1](#_Toc231769247)

[1.2 Data scan acquisitions	1](#_Toc231769248)

[1.3 Data pre-processing	2](#_Toc231769249)

[1.4 Construction of SCN and threshold selection	3](#_Toc231769250)

[2 Supplementary results	4](#_Toc231769251)

[2.1 The result for the sex-by-group interaction of GM	4](#_Toc231769252)

[2.2 The result for the sex-by-group interaction of ALFF	4](#_Toc231769253)

[2.3 The result of the global topology metrics in SCN	4](#_Toc231769254)

[3 Supplementary Tables	5](#_Toc231769255)

[Table S1 Stage-specific GM volumes patterns in male patients with MDD.	5](#_Toc231769256)

[Table S2 Stage-specific GM volumes patterns in female patients with MDD.	6](#_Toc231769257)

[Table S3 The difference with sex × group interaction in GM volumes.	7](#_Toc231769258)

[Table S4 The sex differences in global topology metrics.	8](#_Toc231769259)

[Table S5 The sex difference in regional topology metrics of degree centrality.	9](#_Toc231769260)

[Table S6 The sex difference in regional metrics of betweenness centrality.	10](#_Toc231769261)

[Table S7 Stage-specific ALFF altered patterns in male patients with MDD.	11](#_Toc231769262)

[Table S8 Stage-specific ALFF altered patterns in female patients with MDD.	12](#_Toc231769263)

[Table S9 The difference with sex × group interaction in ALFF values.	13](#_Toc231769264)

[4 Supplementary Figures	14](#_Toc231769265)

[Figure S1 Stage-specific GM patterns in male and female MDD relative to matched HC.	14](#_Toc231769266)

[Reference:	15](#_Toc231769267)

# **1 Supplementary method**

## **1.1 Data selection**

The inclusion criteria for the patients were as follows: (1) a diagnosis of MDD confirmed using the DSM-IV-TR criteria (1); (2) right-handedness; (3) an education level of junior school or higher; (4) age between 16 and 50 years; (5) being of Han Chinese ethnicity; (6) scores greater 17 than HAMD_17_(2); and (7) scores below five on the Young Mania Rating Scale (YMRS)(3). The exclusion criteria included: (1) any other relevant medical or mental health issues aside from MDD; (2) a history of systemic illness; (3) current pregnancy or breastfeeding; (4) substance abuse or dependence; (5) any physical therapies, such as repetitive transcranial magnetic stimulation, administered within the six months prior to scanning; (6) any contraindications to MRI scanning; and (7) a prior transformation to bipolar disorder during follow-up.

The inclusion criteria for HC were: (1) screened negative for MDD via the DSM-IV-TR nonpatient version of the Structured Clinical Interview; (2) right-handedness; (3) age between 18 and 50 years; (4) an education level of junior high school or higher; (5) being of Han Chinese ethnicity; (6) HAMD_17_ scores less than 7; (7) YMRS scores below 5; (8) no history of neurological or mental illnesses; and (9) no family history of neurological or psychiatric illness. The exclusion criteria for HC were consistent with those for the patient group.

## **1.2 Data scan acquisitions**

Imaging data were acquired using a 3-Tesla Siemens Verio scanner with an eight-channel radio frequency coil at Nanjing Brain Hospital. All participants were positioned within a birdcage head coil, equipped with foam padding to minimize head motion. They were instructed to lie quietly on the examination bed, close their eyes, maintain a fixed head position, refrain from excessive thinking, remain awake, and raise their hands if they experienced discomfort during the scan. Head movements were monitored throughout the scanning process. After the MRI scan, participants were asked whether they had fallen asleep or engaged in excessive thinking.

Routine T1-weighted imaging (T1WI) and T2-weighted imaging (T2WI) were initially acquired for participants without abnormalities on routine MRI; 3D-T1 and resting-state functional MRI (rs-fMRI) scans were performed. The parameters for the 3D-T1 scanning were as follows: slice number = 176, slice thickness = 1 mm, repetition time/echo time (TR/TE) = 1900 ms / 2.48 ms, field of view (FOV) = 250 mm × 250 mm, flip angle = 9°, matrix size = 256 × 256, scan duration = 4 minutes 18 seconds. Subsequently, rs-fMRI scanning was performed using gradient-recalled echo-planar imaging (GRE-EPI) with the following parameters: slice number = 32, slice thickness = 4 mm, TR/TE = 3000 ms / 40 ms, FOV = 240 × 240 mm², flip angle = 90°, matrix size = 64 × 64, number of time points = 133, and scan duration = 6 minutes 45 seconds.

## **1.3 Data pre-processing**

High-spatial-resolution T1-weighted MRI data were processed using standard voxel-based morphometry through the Computational Anatomy Toolbox (CAT12; http://dbm.neuro.unijena.de/cat12/) within the Statistical Parametric Mapping 12 (SPM12; http://www.ffl.ion.ucl.ac.uk/spm/) software. Initially, all images were inspected for artifacts and reoriented to align the image origins at the anterior commissure. Subsequently, the T1-weighted images were normalized to Montreal Neurological Institute (MNI) space, segmented into GM, white matter, and cerebrospinal fluid regions, and resampled to a volumetric image resolution of 1 × 1 × 1 mm³. After verifying data quality and sample homogeneity, the segmented GM images were smoothed using an 8-mm full width at half maximum (FWHM) Gaussian kernel. The smoothed GM images were utilized as the GM volumes for subsequent group comparisons.

The Data Processing Assistant for Resting-State fMRI (DPARSF; http://rfmri.org/DPARSF) was employed to analyze the functional imaging data (4). The first six functional volumes were discarded to allow participants to acclimate to the scanner noise. Next, slice timing and head motion (5) correction were conducted. Various sources of variation were controlled, including head-motion (6) characteristics, averaged global blood-oxygen-level-dependent (BOLD) signals, and mean BOLD signals from the ventricular and white matter regions. Spatial normalization to MNI space was performed with a resampled voxel size of 1 × 1 × 1 mm³. Structural images were standardized to the MNI template (T1-weighted), and the data were smoothed with a 4-mm FWHM Gaussian kernel. Finally, linear detrending was applied to reduce low-frequency drift. A fast Fourier transform was employed to convert the filtered time series of each voxel into the frequency domain. ALFF values were calculated as the average square root over the 0.01-0.08 Hz frequency range for each voxel (7).

## **1.4 Construction of SCN and threshold selection**

To construct SCN, GM volumes were first parcellated into 166 regions of interest using the AAL3 atlas (8). These values were adjusted for TIV and years of education using linear regression. The residualized GM values were then used to construct group-level SCN separately for each subgroup (male MDD, female MDD, male HC, female HC). Specifically, for each group, we computed Pearson's correlation coefficients between the residualized GM values of all possible pairs of the 166 regions, yielding a 166 × 166 correlation matrix.

As per the previous study(9), the sparsity threshold was defined by two primary criteria: 1) more than 90% of nodes must remain connected within the network, and 2) the small-world index must exceed 1.1 for over 95% of the intra-individual SCN comprising each dataset. The minimum is calculated in v2.0 Gretna software (https://www.nitrc.org/projects/gretna/) using the code “gretna_get_rmax (rand (N, N))”, with N = 166, denoting the number of nodes. The maximum was selected when the small-world scalar sigma of the threshold networks was larger than 1.1 for all participants. A comprehensive range of sparsity thresholds (male: 0.14-0.42; female: 0.14-0.50; with a step of 0.01) was selected to binarize individual SCN and to facilitate the case-control comparison of global and nodal network topologies.

# **2 Supplementary results**

## **2.1 The result for the sex-by-group interaction of GM**

For the sex-by-group interaction among the four groups (MDD male, MDD female, HC male, HC female), significant differences in GM volumes were identified in the left MCG and right PoCG at stage 1; the left MCG, right PoCG, right IFGtri, left lingual gyrus, and right angular gyrus at stage 2; the left MCG, right PoCG, right precentral gyrus, and bilateral STG at stage 3; and the left MCG and right lingual gyrus at stage 4 (GRF corrected; voxel size *p* < 0.001; cluster size *p* < 0.05; Table S3).

## **2.2 The result for the sex-by-group interaction of ALFF**

For the sex-by-group interaction among the four groups (male MDD, female MDD, healthy male, healthy female), significant differences in ALFF values across the four groups were identified in the left MCG, the left MTG, the right calcarine at stage 1; the right SFG pars orbital, the left lingual gyrus, and the bilateral angular at stage 2; the left MCG, the left PoCG, the right precentral gyrus, and the right angular gyrus at stage 3; the left supplementary motor area and the left precentral gyrus at stage 4 (GRF corrected; voxel size *p* < 0.001; cluster size *p* < 0.05; Table S9).

## **2.3 The result of the** **global topology metrics in SCN**

In terms of global topology metrics, male patients with MDD showed decreased gamma (*p* < 0.001, FDR corrected) and sigma (*p* < 0.001, FDR corrected). No differences in the male group were observed in clustering coefficients, characteristic path length, lambda, and global or local efficiency. Conversely, female patients with MDD demonstrated reductions in clustering coefficients (*p* < 0.001, FDR corrected), gamma (*p* < 0.001, FDR corrected), sigma (*p* < 0.001, FDR corrected), and global efficiency (*p* = 0.023, FDR corrected), with no differences related to the female group in characteristic path length, lambda, or local efficiency (Table S4).

# **3 Supplementary Tables**

## **Table S1 Stage-specific GM volumes patterns in male patients with MDD.**

| Duration | **Male** | MNI | | | Cluster size | *t*-value |
| --- | --- | --- | --- | --- | --- | --- |
|  |  | x | y | z |  |  |
| ***0-3 months*** | MCG_L | -10 | -41 | 32 | 1757 | -6.7682 |
|  | Postcentral_R | 20 | -40 | 62 | 869 | -3.735 |
|  | Lingual _L | -26 | -85 | -8 | 1076 | -4.5684 |
| ***4-12 months*** | MCG_L | -13 | -42 | 35 | 2919 | -5.7672 |
|  | Postcentral_R | 63 | -14 | 29 | 1273 | -3.8959 |
|  | Lingual _L | -23 | -68 | -5 | 4474 | -4.1908 |
|  | Lingual _R | 25 | -72 | -12 | 2520 | -4.1831 |
| ***1-3 years*** | MCG_L | -10 | -35 | 33 | 2155 | -5.607 |
|  | IFGtri_L | -44 | 20 | 23 | 1019 | -3.9911 |
|  | Postcentral_L | -35 | -25 | 44 | 2852 | -3.9909 |
|  | Postcentral_R | 53 | -17 | 40 | 9334 | -4.6876 |
|  | Lingual_R | 23 | -59 | -8 | 2110 | -5.1775 |
|  | STG_L | -62 | -34 | 14 | 1292 | -4.2683 |
| ***>3 years*** | MCG_L | -10 | -36 | 33 | 1909 | -5.8009 |
|  | IFGorb_L | -34 | 40 | -11 | 660 | -3.8296 |
|  | IFGtri _L | -47 | 35 | 8 | 1737 | -3.8153 |
|  | IFGtri_R | 44 | 35 | 22 | 229 | -3.4865 |
|  | MFGorb_R | 35 | 50 | -8 | 525 | -3.9006 |
|  | Lingual_L | -21 | -48 | -11 | 4491 | -4.0783 |
|  | Lingual_R | 16 | -61 | -5 | 14282 | -5.3721 |

***Abbreviations:*** MDD, major depressive disorder; GM, gray matter; MNI, Montreal Neurological Institute; MCG, middle cingulum gyrus; IFG, inferior frontal gyrus; STG, superior temporal gyrus; MFG, middle frontal gyrus; Tri, triangularis; Orb, orbitalis; L, left; R, right.

x, y, z are the coordinates of the primary peak locations in the MNI space.

*t* is obtained by two-sample two-tailed *t*-test (GRF corrected; voxel size *p* < 0.001; cluster size *p* < 0.05)

| Duration | **Female** | MNI | | | Cluster size | *t*-value |
| --- | --- | --- | --- | --- | --- | --- |
|  |  | x | y | z |  |  |
| ***0-3 month*** | ITG_L | -37 | -8 | -32 | 429 | -4.7538 |
|  | Insula_L | -35 | -11 | 22 | 841 | -5.0655 |
|  | Insula_R | 38 | -8 | 19 | 999 | -4.4107 |
| ***4-12 months*** | ITG_L | -40 | -5 | -32 | 406 | -5.5638 |
|  | ITG_R | 44 | -53 | -8 | 908 | -5.232 |
|  | MTG_L | -67 | -17 | -11 | 1127 | -4.6483 |
|  | MTG_R | 62 | -59 | -23 | 2360 | 5.6689 |
|  | STG_R | 71 | -7 | -5 | 855 | 5.584 |
|  | IFGorb_L | -13 | 13 | -26 | 212 | 4.9051 |
|  | IFGtri_R | 59 | 5 | -41 | 4016 | 6.9723 |
|  | IFGtri_L | -49 | 46 | 10 | 355 | -4.5561 |
|  | MFG_R | 35 | 47 | 7 | 734 | -4.7352 |
|  | SFG_L | -13 | 49 | 19 | 1845 | -5.6858 |
|  | Putamen_L | -16 | 7 | 2 | 311 | 4.9316 |
|  | Insula_L | -38 | -11 | 22 | 1928 | -6.4909 |
|  | Insula_R | 35 | -14 | 22 | 938 | -5.3892 |
|  | Hippocampus_L | -25 | -32 | -2 | 9584 | -6.9605 |
|  | Hippocampus_R | 17 | -7 | -14 | 2182 | -6.24 |
|  | Fusiform_R | 34 | -2 | -38 | 573 | -5.2005 |
| ***1-3 years*** | ITG_L | -59 | -14 | -23 | 240 | -4.4941 |
|  | ITG_R | 50 | -17 | -26 | 2032 | -5.0465 |
|  | MTG_R | 62 | -56 | -23 | 4758 | 6.4601 |
|  | STG_R | 71 | -1 | 18 | 471 | 6.0203 |
|  | IFGtri_R | 68 | 2 | 5 | 4844 | 6.435 |
|  | IFGorb_L | -29 | 37 | -8 | 245 | -5.5457 |
|  | SFG_L | -13 | 49 | 19 | 825 | -5.1946 |
|  | SPG_R | 47 | -56 | 62 | 247 | 5.4465 |
|  | Pallidum_L | -19 | 1 | -4 | 994 | 5.9002 |
|  | Insula_L | -29 | 25 | 13 | 433 | -4.6617 |
|  | Insula_R | 38 | -11 | 22 | 649 | -5.7039 |
|  | Hippocampus_L | -17 | -13 | -14 | 8503 | -6.3189 |
|  | Hippocampus_R | 17 | -7 | -14 | 224 | -5.7666 |
|  | Calcarine_L | 2 | -101 | -7 | 570 | 5.8352 |
|  | Precuneus_L | -13 | -47 | 7 | 704 | -5.0527 |
|  | Occipital_Mid_R | 26 | -89 | 10 | 208 | -3.8837 |
|  | SupraMarginal_R | 62 | -26 | 35 | 242 | -3.8939 |
| ***>3 years*** | ITG_L | -49 | -20 | -23 | 14733 | -6.1741 |
|  | ITG_R | 48 | -20 | -23 | 6336 | -6.6932 |
|  | MTG_L | -59 | -32 | 4 | 269 | -3.8073 |
|  | MTG_R | 62 | -53 | -26 | 2155 | 5.7195 |
|  | STG_L | -43 | -13 | -10 | 1716 | -4.6876 |
|  | STG_R | 56 | -32 | 7 | 2691 | -4.8004 |
|  | STP_L | -55 | 10 | -5 | 549 | -4.0021 |
|  | STP_R | 65 | 8 | -14 | 536 | 5.3772 |
|  | IFGTri_R | 59 | 20 | 38 | 616 | 5.2149 |
|  | MFGorb_L | -43 | 58 | -1 | 2933 | -5.2151 |
|  | MFGorb_R | 46 | 55 | -10 | 1888 | -4.9562 |
|  | SFG_R | 17 | 4 | 53 | 6603 | -5.9983 |
|  | Precentral_L | -35 | -23 | 55 | 512 | -4.5158 |
|  | Precentral_R | 47 | -8 | 40 | 208 | -4.2027 |
|  | SPG_R | 23 | -53 | 56 | 683 | -4.3755 |
|  | Caudate_L | 5 | 22 | -2 | 1275 | -5.4055 |
|  | Insula_R | 35 | -14 | 22 | 3383 | -6.0504 |
|  | Insula_L | -32 | -17 | 22 | 2964 | -5.925 |
|  | Thalamus_R | 11 | -16 | -5 | 365 | 5.7168 |
|  | Precuneus_L | -16 | -56 | 13 | 4245 | -5.2161 |
|  | Fusiform_R | 23 | -62 | -5 | 1204 | -4.5837 |
|  | Precuneus_R | 19 | -50 | 13 | 277 | -4.8267 |
|  | SupraMarginal_R | 67 | -17 | 40 | 260 | -4.0889 |

## **Table S2 Stage-specific GM volumes patterns in female patients with MDD.**

***Abbreviations:*** MDD, major depressive disorder; GM, gray matter; MNI, Montreal Neurological Institute; ITG, inferior temporal gyrus; MTG, middle temporal gyrus; STG, superior temporal gyrus; STP, superior temporal pole; IFG, inferior frontal gyrus; MFG, middle frontal gyrus; SFG, superior frontal gyrus; SPG, superior parietal gyrus; MOG, middle occipital gyrus; Tri, triangularis; Orb, orbitalis; L, left; R, right.

x, y, z are the coordinates of the primary peak locations in the MNI space

*t* is obtained by two-sample two-tailed *t*-test (GRF corrected; voxel size *p* < 0.001; cluster size *p* < 0.05)

## **Table S3 The difference with sex × group interaction in GM volumes.**

| Duration | **Sex × Group** | MNI | | | Cluster size | *F*-value |
| --- | --- | --- | --- | --- | --- | --- |
|  |  | x | y | z |  |  |
| ***0-3 months*** | MCG_L | -12 | -40 | 34 | 1444 | 44.3496 |
|  | Postcentral_R | 19 | -42 | 61 | 1111 | 19.3863 |
| ***4-12 months*** | MCG_L | -11 | -40 | 34 | 372 | 21.0313 |
|  | Postcentral_R | 68 | 2 | 7 | 54 | 14.4919 |
|  | Lingual_L | -10 | -101 | -17 | 211 | 17.1643 |
|  | IFGtri_R | 65 | 23 | 15 | 64 | 13.8951 |
|  | Angular_R | 40 | -52 | 31 | 70 | 21.5358 |
| ***1-3 years*** | MCG_L | -11 | -37 | 31 | 924 | 28.0246 |
|  | Postcentral_R | 71 | -17 | 20 | 2941 | 26.5771 |
|  | Precentral_R | 40 | -20 | 40 | 451 | 16.3011 |
|  | STG_L | -59 | -29 | 10 | 407 | 14.6049 |
|  | STG_R | 65 | 2 | 4 | 83 | 16.7313 |
| ***>3 years*** | MCG_L | -11 | -37 | 31 | 229 | 19.3631 |
|  | Lingual_R | 14 | -62 | -1 | 88 | 13.9658 |

***Abbreviations:*** GM, gray matter; MNI, Montreal Neurological Institute; MCG, middle cingulum gyrus; IFG, inferior frontal gyrus; STG, superior temporal gyrus; Tri, triangularis; L, left; R, right.

x, y, z are the coordinates of the primary peak locations in the MNI space.

*F* is obtained by two-way ANOVA (GRF corrected; voxel size *p* < 0.001; cluster size *p* < 0.05).

| Global metrics | | | MDD (Male/Female=326/485) | HC (Male/Female=138/221) | *p* |
| --- | --- | --- | --- | --- | --- |
| Clustering coefficient (*Cp*) | Male | Original | 0.279 | 0.269 | 0.299 |
|  |  | Random-null | 0.274±0.003 | 0.270±0.005 |  |
|  | Female | Original | 0.352 | 0.331 | <0.001 |
|  |  | Random-null | 0.350±0.002 | 0.347±0.003 |  |
| Characteristic path length (*Lp*) | Male | Original | 0.837 | 0.827 | 0.808 |
|  |  | Random-null | 0.885±0.014 | 0.880±0.027 |  |
|  | Female | Original | 1.032 | 0.961 | 0.104 |
|  |  | Random-null | 1.096±0.014 | 1.085±0.030 |  |
| Gamma (*γ*) | Male | Original | 0.738 | 0.925 | <0.001*** |
|  |  | Random-null | 0.599±0.016 | 0.603±0.039 |  |
|  | Female | Original | 0.901 | 1.125 | <0.001*** |
|  |  | Random-null | 0.698±0.014 | 0.705±0.030 |  |
| Lambda (*λ*) | Male | Original | 0.466 | 0.462 | 0.887 |
|  |  | Random-null | 0.477±0.011 | 0.471±0.020 |  |
|  | Female | Original | 0.582 | 0.548 | 0.112 |
|  |  | Random-null | 0.610±0.007 | 0.603±0.014 |  |
| Sigma (*σ*) | Male | Original | 0.575 | 0.738 | <0.001*** |
|  |  | Random-null | 0.464±0.017 | 0.523±0.000 |  |
|  | Female | Original | 0.693 | 0.919 | <0.001*** |
|  |  | Random-null | 0.520±0.012 | 0.526±0.000 |  |
| Global efficiency | Male | Original | 0.189 | 0.198 | 0.158 |
|  |  | Random-null | 0.178±0.003 | 0.180±0.004 |  |
|  | Female | Original | 0.243 | 0.257 | 0.023* |
|  |  | Random-null | 0.387±0.003 | 0.230±0.004 |  |
| Local  efficiency | Male | Original | 0.316 | 0.317 | 0.965 |
|  |  | Random-null | 0.309±0.003 | 0.307±0.006 |  |
|  | Female | Original | 0.396 | 0.389 | 0.181 |
|  |  | Random-null | 0.388±0.002 | 0.387±0.003 |  |

## **Table S4 The sex differences in global topology metrics.**

***Abbreviations:*** MDD, major depressive disorder; HC, health controls.

Random-null data were presented as the range of minimum-maximum (mean ± SD).

*p* is obtained by two-sample *t*-test (FDR corrected; **p* < 0.05, ** *p* < 0.01; *** *p* < 0.001 voxel size *p* < 0.001)

## **Table S5 The sex difference in regional topology metrics of degree centrality.**

| Regional metrics | | | MDD (Male/Female=326/485) | HC (Male/Female=138/221) | *p* |
| --- | --- | --- | --- | --- | --- |
| *Male* | OFCmed_L | Original | 22.765 | 13.575 | <0.001 |
|  | OFCpost_L | Original | 23.635 | 14.5 | 0.002 |
|  | OFCpost_R | Original | 23.675 | 14.415 | <0.001 |
|  | MCG_R | Original | 31.795 | 21.04 | <0.001 |
|  | Lingual_R | Original | 30.2 | 20.965 | <0.001 |
|  | Fusiform_L | Original | 31.87 | 20.94 | <0.001 |
|  | Fusiform_R | Original | 30.985 | 21.51 | <0.001 |
|  | NAc_L | Original | 23.77 | 11.445 | 0.002 |
|  | NAc_R | Original | 19.625 | 8.82 | 0.002 |
| *Female* | SFGdl_L | Original | 36.77 | 30.53 | <0.001 |
|  | SFGdl_R | Original | 37.125 | 26.745 | <0.001 |
|  | MFG_L | Original | 35 | 27.495 | <0.001 |
|  | MFG_R | Original | 33.055 | 28.3 | <0.001 |
|  | IFGorb_R | Original | 30.99 | 23.39 | <0.001 |
|  | SFGmed_R | Original | 33.075 | 26.245 | <0.001 |
|  | PFCvm_R | Original | 32.145 | 25.395 | <0.001 |
|  | OFCpost_L | Original | 28.75 | 19.875 | <0.001 |
|  | Temporal_Mid_R | Original | 37.635 | 28.45 | <0.001 |
|  | Temporal_Inf_L | Original | 30.455 | 23.695 | 0.002 |
|  | Temporal_Inf_R | Original | 30.18 | 16.615 | <0.001 |
|  | ACC_pre_R | Original | 33.035 | 25.49 | 0.001 |

***Abbreviations:*** MDD, major depressive disorder; HC, health controls; OFC, orbitofrontal cortex; MCG, middle cingulum gyrus; NAc, nucleus accumbens; SFG, superior frontal gyrus; MFG, medial frontal gyrus; IFG, inferior frontal gyrus; PFC, prefrontal cortex; ACC, anterior cingulate cortex; med, medial; post, posterior; mid, middle; dl, dorsolateral; orb, orbitalis; vm, ventromedial; inf, inferior; pre, pregenual; L, left; R, right.

*p* is obtained by two-sample *t*-test (FDR corrected; *p* < 0.05)

| Regional metrics | | | MDD (Male/Female=326/485) | HC (Male/Female=138/221) | *p* |
| --- | --- | --- | --- | --- | --- |
| Male | IFGoper_R | Original | 2.01 | 128.02 | <0.001 |
|  | IFGorb_L | Original | 20.74 | 90.31 | <0.001 |
|  | SFGmed_L | Original | 35.64 | 189.53 | <0.001 |
|  | OFCant_R | Original | 8.95 | 180.66 | <0.001 |
|  | Cuneus_L | Original | 20.34 | 67.50 | <0.001 |
|  | Postcentral_R | Original | 4.19 | 34.50 | <0.001 |
| Female | IFGoper_L | Original | 9.32 | 22.27 | <0.001 |
|  | OFCpost_R | Original | 28.66 | 98.92 | <0.001 |
|  | IPG_R | Original | 7.41 | 87.19 | <0.001 |
|  | STG_R | Original | 173.01 | 466.33 | <0.001 |
|  | STP_L | Original | 208.58 | 428.75 | <0.001 |
|  | STP_R | Original | 106.99 | 206.87 | <0.001 |

## **Table S6 The sex difference in regional metrics of betweenness centrality****.**

***Abbreviations:*** MDD, major depressive disorder; HC, health controls; IFG, inferior frontal gyrus; SFG, superior frontal gyrus; OFC, orbitofrontal cortex; IPG, inferior parietal gyrus; STG, superior temporal gyrus; STP, superior temporal pole; oper, opercular; post, posterior; inf, inferior; L, left; R, right.

*p* is obtained by two-sample *t*-test (FDR corrected; *p* < 0.05)

## **Table S7 Stage-specific ALFF altered patterns in male patients with MDD.**

| Duration | **Male** | MNI | | | Cluster size | *t*-value |
| --- | --- | --- | --- | --- | --- | --- |
|  |  | x | y | z |  |  |
| ***0-3 months*** | MCG_L | -7 | -42 | 30 | 283 | -4.4689 |
|  | Angular_L | -45 | -51 | 23 | 789 | -5.2372 |
|  | Angular_R | 51 | -57 | 30 | 345 | -5.0968 |
|  | Precuneus_L | 6 | -47 | 22 | 577 | -4.8849 |
|  | Precuneus_R | 1 | -67 | 40 | 100 | -4.2046 |
|  | SOG_R | 31 | -80 | 47 | 158 | 4.2391 |
| ***4-12 months*** | MCG_L | -9 | -42 | 30 | 233 | -4.3074 |
|  | MFG_R | 61 | 12 | 36 | 111 | 4.0396 |
|  | Precentral_R | 54 | 0 | 47 | 322 | 4.9738 |
|  | SPG_R | 29 | -75 | 50 | 223 | 3.9514 |
|  | MOG_R | 37 | -89 | 10 | 368 | 4.3053 |
|  | SOG_R | 19 | -88 | 35 | 128 | 4.3333 |
|  | Angular_L | -43 | -50 | 23 | 647 | -5.0302 |
|  | Angular_R | 49 | -55 | 27 | 260 | -5.5399 |
|  | Fusiform_R | 23 | -59 | -17 | 107 | -4.4738 |
|  | Lingual_R | 11 | -78 | -7 | 109 | -4.0587 |
| ***1-3 years*** | MTG_R | 52 | -52 | 1 | 123 | -4.765 |
|  | Precuneus_L | -6 | -68 | 50 | 324 | -4.8242 |
|  | IPG_R | 45 | -53 | 41 | 143 | -4.7252 |
|  | MOG_R | 37 | -83 | 27 | 139 | 4.1445 |
|  | Angular_R | 50 | -56 | 32 | 188 | -4.8646 |
| ***>3 years*** | SFG_L | -20 | 12 | 50 | 130 | -5.2875 |
|  | Angular_L | -40 | -63 | 25 | 133 | -4.8058 |
|  | MOG_R | 33 | -95 | 3 | 103 | 4.5742 |

***Abbreviations:*** MDD, major depressive disorder; GM, gray matter; MNI, Montreal Neurological Institute; MCG, middle cingulum gyrus; SOG, superior occipital gyrus; MFG, middle frontal gyrus; SPG, superior parietal gyrus; MOG, middle occipital gyrus, MTG, middle temporal gyrus; IPG, inferior parietal gyrus; SFG, superior frontal gyrus; L, left; R, right.

x, y, z are the coordinates of the primary peak locations in the MNI space.

*t* is obtained by two-sample two-tailed *t*-test (GRF corrected; voxel size *p* < 0.001; cluster size *p* < 0.05)

## **Table S8 Stage-specific ALFF altered patterns in female patients with MDD.**

| Duration | **Female** | MNI | | | Cluster size | *t*-value |
| --- | --- | --- | --- | --- | --- | --- |
|  |  | x | y | z |  |  |
| ***0-3 months*** | ITG_L | -52 | -62 | -6 | 201 | -4.8837 |
| ***4-12 months*** | STG_L | -40 | -39 | 15 | 284 | 5.2051 |
|  | MOG_R | 42 | -65 | 22 | 118 | -4.1074 |
|  | IOG_R | 51 | -74 | -7 | 155 | 4.5691 |
|  | Hippocampus_L | -14 | -41 | 10 | 106 | 6.4283 |
| ***1-3 years*** | STG_L | -41 | -39 | 15 | 159 | 4.6157 |
|  | Angular_L | -43 | -56 | 42 | 159 | -4.9466 |
| ***>3 years*** | ITG _R | 66 | -22 | -23 | 213 | 5.0134 |
|  | MTG_R | 42 | -62 | 21 | 201 | -4.5836 |
|  | IFGtri_R | 51 | 42 | 23 | 123 | 4.5355 |
|  | MFG_R | 42 | 40 | 39 | 215 | 4.1853 |
|  | Precentral_L | -32 | -18 | 72 | 124 | 3.8247 |
|  | IPG_R | 43 | -52 | 38 | 342 | -5.943 |
|  | Caudate_R | 12 | 18 | -5 | 177 | 4.196 |
|  | Hippocampus_L | -14 | -40 | 6 | 220 | 5.7826 |
|  | Hippocampus_R | 28 | -37 | -4 | 118 | 4.4617 |
|  | ParaHippocampal_R | 18 | -31 | -16 | 210 | 4.8768 |
|  | IOG_R | 30 | -101 | -8 | 713 | 5.5102 |
|  | MOG_R | 36 | -89 | 18 | 279 | 4.6435 |

***Abbreviations:*** MDD, major depressive disorder; GM, gray matter; MNI, Montreal Neurological Institute; ITG, inferior temporal gyrus; MTG, middle temporal gyrus; STG, superior temporal gyrus; MOG, middle occipital gyrus; IOG, inferior occipital gyrus; IFG, inferior frontal gyrus; MFG, middle frontal gyrus; IPG, inferior parietal gyrus; Tri, triangularis; L, left; R, right.

x, y, z are the coordinates of the primary peak locations in the MNI space

*t* is obtained by two-sample two-tailed *t*-test (GRF corrected; voxel size *p* < 0.001; cluster size *p* < 0.05)

| Duration | **Sex × Group** | MNI | | | Cluster size | *F*-value |
| --- | --- | --- | --- | --- | --- | --- |
|  |  | x | y | z |  |  |
| ***0-3 months*** | MCG_L | -7 | -42 | 33 | 259 | 20.6117 |
|  | MTG_L | -44 | -51 | 18 | 33 | 13.274 |
|  | Calcarine_R | 15 | -95 | 2 | 124 | 22.106 |
| ***4-12 months*** | SFGorb_R | 11 | 32 | -23 | 41 | 17.9217 |
|  | Lingual_L | -12 | -83 | 1 | 39 | 15.0905 |
|  | Angular_L | -45 | -50 | 30 | 90 | 20.8306 |
|  | Angular_R | 47 | -55 | 27 | 83 | 20.7149 |
| ***1-3 years*** | MCG_L | -8 | -42 | 34 | 34 | 17.4515 |
|  | Postcentral_L | -46 | -15 | 37 | 30 | 14.0528 |
|  | Precentral_R | 48 | -6 | 36 | 40 | 14.9642 |
|  | Angular_R | 50 | -56 | 32 | 28 | 16.9844 |
| ***>3 years*** | SMA_L | 1 | 20 | 65 | 89 | 22.4003 |
|  | Precentral_L | -37 | -6 | 41 | 32 | 17.9718 |

## **Table S9 The difference with sex × group interaction in ALFF values.**

***Abbreviations:*** GM, gray matter; MNI, Montreal Neurological Institute; MCG, middle cingulum gyrus; MTG, middle temporal gyrus; SFG, superior frontal gyrus; SMA, supplementary motor area; Orb, orbitalis; L, left; R, right.

x, y, z are the coordinates of the primary peak locations in the MNI space.

*F* is obtained by two-way ANOVA (GRF corrected; voxel size *p* < 0.001; cluster size *p* < 0.05).

# **4 Supplementary Figures**

## **Figure S1 Stage-specific GM patterns in male and female MDD relative to matched HC.**

A. Male MDD vs. male HC. These regions were selected as representative regions showing significant GM volume differences between male MDD patients and matched HC at one or more disease stages (two-sample t-test, GRF-corrected voxel-level *p* < 0.005, cluster-level *p* < 0.00625 after Bonferroni correction). B. Female MDD vs. female HC. These regions were selected using the same criteria as males.

# **Reference:**

1.	APA: Diagnostic and Statistical Manual of Mental Disorders, 4th Ed. 2000.

2.	Hamilton M. Development of a rating scale for primary depressive illness. The British journal of social and clinical psychology. 1967;6(4):278-96.

3.	Young RC, Biggs JT, Ziegler VE, Meyer DA. A rating scale for mania: reliability, validity and sensitivity. The British journal of psychiatry : the journal of mental science. 1978;133:429-35.

4.	Chao-Gan Y, Yu-Feng Z. DPARSF: A MATLAB Toolbox for "Pipeline" Data Analysis of Resting-State fMRI. Front Syst Neurosci. 2010;4:13.

5.	Power JD, Barnes KA, Snyder AZ, Schlaggar BL, Petersen SE. Spurious but systematic correlations in functional connectivity MRI networks arise from subject motion. NeuroImage. 2012;59(3):2142-54.

6.	Friston KJ, Williams S, Howard R, Frackowiak RS, Turner R. Movement-related effects in fMRI time-series. Magn Reson Med. 1996;35(3):346-55.

7.	Zang YF, He Y, Zhu CZ, Cao QJ, Sui MQ, Liang M, et al. Altered baseline brain activity in children with ADHD revealed by resting-state functional MRI. Brain & development. 2007;29(2):83-91.

8.	Rolls ET, Huang CC, Lin CP, Feng J, Joliot M. Automated anatomical labelling atlas 3. NeuroImage. 2020;206:116189.

9.	Wang X, Xia Y, Yan R, Wang H, Sun H, Huang Y, et al. The relationship between disrupted anhedonia-related circuitry and suicidal ideation in major depressive disorder: A network-based analysis. NeuroImage Clinical. 2023;40:103512.
